# Nomenclature for kidney function and disease: executive summary and glossary from a Kidney Disease: Improving Global Outcomes (KDIGO) consensus conference

**DOI:** 10.1007/s40620-020-00773-6

**Published:** 2020-07-16

**Authors:** Andrew S. Levey, Kai-Uwe Eckardt, Nijsje M. Dorman, Stacy L. Christiansen, Michael Cheung, Michel Jadoul, Wolfgang C. Winkelmayer

**Affiliations:** 1 Division of Nephrology, Tufts Medical Center, 850 Washington Street, Box 391, Boston, MA 02111 USA; 2 Department of Nephrology and Medical Intensive Care, Charité—Universitätsmedizin Berlin, Augustenburger Platz 1, 13353 Berlin, Germany; 3 American Journal of Kidney Diseases, Philadelphia, USA; 4 Journal of the American Medical Association, Chicago, USA; 5 Kidney Disease: Improving Global Outcomes (KDIGO), Brussels, Belgium; 6 Cliniques Universitaires Saint Luc, Université Catholique de Louvain, Brussels, Belgium; 7 Selzman Institute for Kidney Health, Section of Nephrology, Department of Medicine, Baylor College of Medicine, Houston, TX USA

A primary obligation of medical journals is the responsible, professional, and expeditious delivery of knowledge from researchers and practitioners to the wider community [1]. The task of journal editors, therefore, rests not merely in selecting what to publish, but in large measure judging how it can best be communicated. The challenge of improving descriptions of kidney function and disease in medical publishing was the impetus for a Kidney Disease: Improving Global Outcomes (KDIGO) Consensus Conference held in June 2019. The conference goals included standardizing and refining kidney-related nomenclature used in English-language scientific articles and developing a glossary that can be used by journals [2].

The rationale for the conference was that the worldwide burden of kidney disease is rising, but public awareness remains limited, underscoring the need for effective communication by stakeholders in the kidney health community [3–6]. Despite this need, the nomenclature for describing kidney function and disease lacks uniformity and clarity. Two decades ago, a survey of hundreds of published articles and meeting abstracts reported a broad array of overlapping, confusing terms for chronic kidney disease (CKD) and advocated adoption of unambiguous terminology [7]. Nevertheless, terms flagged by that analysis as problematic, such as “chronic renal failure” and “pre-dialysis,” still appear in current-day publications. A coherent, shared nomenclature could improve communication at all levels, to not only foster better appreciation of the burden of disease but also aid understanding of how patients feel about their disease, allow more effective communication between kidney disease specialists and other clinicians, advance more straightforward comparison and integration of datasets, enable better recognition of gaps in knowledge for future research, and facilitate more comprehensive public health policies for acute and chronic kidney disease.

Developing consistent, patient-centered, and precise descriptions of kidney function and disease in the scientific literature is an important objective to align communication in clinical practice, research, and public health. Although some terms have been in use for decades, the increased exchange of information among stakeholders makes it timely to revisit nomenclature in order to ensure consistency. The goal is to facilitate communication within and across disciplines and between practitioners and patients, with the ultimate hope of improving outcomes through consistency and precision.

Attendees at the conference included editors of kidney subspecialty journals, kidney subspecialty editors at general medical journals and journals from other subspecialties, experienced authors of clinical kidney health research, and patients. Guiding principles of the conference were that the revised nomenclature should be patient-centered, precise, and consistent with nomenclature used in the KDIGO guidelines. The discussion focused on general description of acute and chronic kidney disease and kidney measures, rather than specific kidney diseases and particular measures of function and structure. Classifications of causes of kidney disease and procedures, performance measures, and outcome metrics for dialysis and transplantation were considered beyond the scope of discussion.

As described in detail in the conference report [8] the meeting attendees reached general consensus on the following recommendations: (1) to use “kidney” rather than “renal” or “nephro-” when referring to kidney disease and kidney function; (2) to use “kidney failure” with appropriate descriptions of presence or absence of symptoms, signs, and treatment rather than “end-stage kidney disease”; (3) to use the KDIGO definition and classification of acute kidney diseases and disorders (AKD) and acute kidney injury (AKI) rather than alternative descriptions to define and classify the severity of these; (4) to use the KDIGO definition and classification of CKD rather than alternative descriptions to define and classify it; and (5) to use specific kidney measures, such as albuminuria or decreased glomerular filtration rate, rather than “abnormal” or “reduced” kidney function to describe alterations in kidney structure and function (Table 1). Accordingly, the proposed glossary contains five corresponding sections, and comprises specific items for which there was general agreement among the conference participants (https://kdigo.org/conferences/nomenclature/; Table 2) [8]. For each section, the glossary includes preferred terms, abbreviations, descriptions, and terms to avoid, with the acknowledgment that journals may choose which of the recommendations to implement, and that journal style will dictate when and how to abbreviate terms to be consistent with nomenclature for other diseases.

**Table 1:** Key takeaways from the conference

Use the term “kidney” rather than “renal” to describe kidney function and kidney disease. In English, the terms renal and kidney are still used interchangeably, resulting in different acronyms describing the same condition or status (e.g., ESRD/ESKD and RRT/KRT). It is more likely that patients and the public would understand the terms incorporating the more familiar noun “kidney,” rather than the less familiar adjective “renal,” which is derived from Latin and is labeled as technical in some dictionaries. Although writing guides may generally favor using an appropriate adjective over a noun as a modifier, there are high-profile precedents for the use of kidney as a modifier, such as AKI, CKD, and NIDDK (National Institute of Diabetes and Digestive and Kidney Diseases)
Avoid the term “end-stage.” Although rooted in US law, the term is not patient sensitive, may connote a stigma, and may discourage advocacy. In the US, ESRD (ESKD) is a synonym for receipt of KRT. However, KRT is a treatment rather than a disease. The term “kidney failure,” which is defined as GFR < 15 ml/min per 1.73 m^2^ or treatment by dialysis, is as comprehensive as “ESRD/ESKD,” without suffering from its limitations
Improve characterization of the full spectrum of kidney failure. Although all patients with kidney failure have GFR < 15 ml/min per 1.73 m^2^ or are undergoing treatment by dialysis, the severity of symptoms varies greatly. We lack terms to describe the severity of symptoms and signs, and yet they are indications for initiating KRT. There are also no common patient-reported outcome measures to describe severity. The term “kidney failure” in a chronic setting is defined as > 3 months, whereas in an acute setting (i.e., AKI stage 3), it is reserved for a duration of ≤ 3 months. Kidney failure could be further classified according to patient-reported outcomes (symptoms)
Use more-descriptive terms for treatments for kidney failure. Many patients with kidney failure do not undergo KRT. The terms “treated” vs. “untreated” have been used, but this is not consistent with the idea that supportive care is indeed treatment. Furthermore, in some cases, patients choose supportive care rather than KRT; in other cases, they do not have a choice because of lack of insurance or lack of availability. Finally, some patients may not be under the care of a physician at all
Avoid the use of “chronic kidney disease (CKD)” as a synonym for “GFR < 60 ml/min per 1.73 m^2^.” CKD includes markers of kidney damage or GFR < 60 ml/min per 1.73 m^2^ for > 3 months, so ascertainment of GFR without assessment for markers of kidney damage is insufficient for classification of CKD status when GFR > 60 ml/min per 1.73 m^2^. If chronicity is not documented, it can be inferred on the basis of corroborative clinical data or presumed in the absence of clinical data to the contrary
Avoid the use of “acute kidney injury (AKI)” as a synonym for “acute kidney diseases and disorders (AKD).” AKD refers to kidney diseases and disorders with a duration of ≤ 3 months, whereas AKI refers to kidney diseases and disorders with onset within 1 week
Use “CKD GFR and albuminuria categories” and “AKI stages” to describe disease severity, rather than employing ill-defined terms such as “mild,” “moderate,” “severe,” and “advanced”
Use the terms “GFR categories” and “albuminuria categories” rather than “CKD stages” when describing the level of GFR and albuminuria in populations either without CKD or without ascertainment of both GFR and albuminuria
Use the term “risk categories” to describe combinations of the G (GFR) and A (albuminuria) categories from the KDIGO heat map (see Supplementary Figure S1)
Use specific terms, such as “GFR,” “tubular secretion,” “tubular reabsorption,” “albuminuria,” and “proteinuria,” rather than general terms, such as “abnormal” or “reduced” kidney function, damage, or injury, when possible. Because kidney function comprises several functional categories, including excretory, endocrine, and metabolic functions, it should be described as specifically as possible. GFR is closely linked with the excretory function, but it should not be used as a synonym, because tubular reabsorption and excretion also contribute to excretory function
When referring to “decreased or decreasing GFR,” avoid the use of different, poorly defined terms such as “impaired kidney function,” “renal insufficiency,” “renal dysfunction,” “renal impairment,” “worsening kidney function,” and “kidney function decline”
When referring to GFR, use descriptive abbreviations (mGFR for measured GFR and eGFR for estimated GFR, with specific notation based on the endogenous filtration markers used (e.g., eGFR_cr_, eGFR_cys_, and eGFR_cr-cys_). Additional detail can be given in the methods. For mGFR, the methods should describe the exogenous filtration marker (e.g., inulin, iothalamate, iohexol) and clearance method (urinary clearance, plasma clearance). For eGFR, the methods should describe the estimating equation used (CKD-EPI; MDRD Study)
Avoid referring to “albuminuria” or “proteinuria” as “decreased kidney function.” Albuminuria and proteinuria are markers of kidney damage, rather than measures of kidney function
When referring to albuminuria or proteinuria, avoid the terms “microalbuminuria” and “macroalbuminuria/clinical proteinuria.” Use the terms “moderately increased” or “severely increased” instead
When referring to albuminuria and proteinuria, use descriptive abbreviations, such as “urine albumin or protein excretion rates (AER and PER)” and “urine albumin–creatinine or protein–creatinine ratios (ACR and PCR)”

ACR, albumin-creatinine ratio; AER, albumin excretion rate; AKD, acute kidney diseases and disorders; AKI, acute kidney injury; CKD, chronic kidney disease; CKD-EPI, CKD Epidemiology Collaboration; eGFR, estimated glomerular filtration rate; eGFR_cr_, estimated glomerular filtration rate derived from creatinine; eGFR_cr-cys_, estimated glomerular filtration rate derived from creatinine and cystatin C; eGFR_cys_, estimated glomerular filtration rate derived from cystatin C; ESKD, end-stage kidney disease; ESRD, end-stage renal disease; GFR, glomerular filtration rate; KDIGO, Kidney Disease: Improving Global Outcomes; KRT, kidney replacement therapy; MDRD, Modification of Diet in Renal Disease; mGFR, measured glomerular filtration rate; NIDDK, National Institute of Diabetes and Digestive and Kidney Diseases; PCR, protein-creatinine ratio; PER, protein excretion rate; RRT, renal replacement therapy; US, United States

**Table 2:**
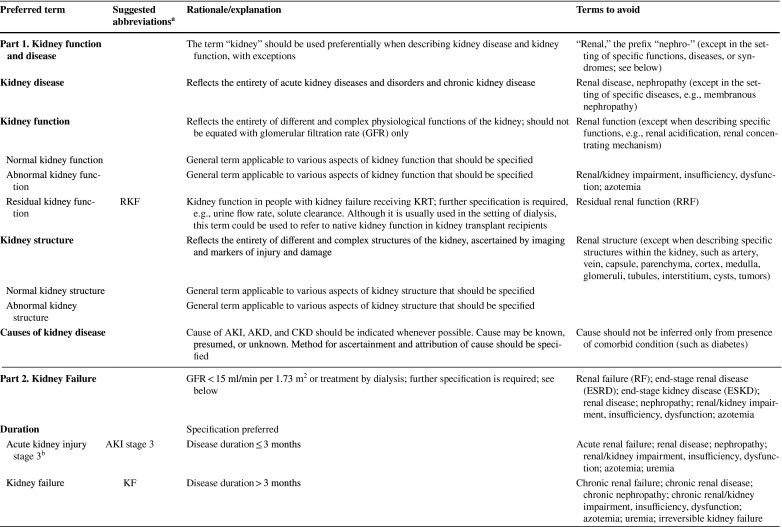

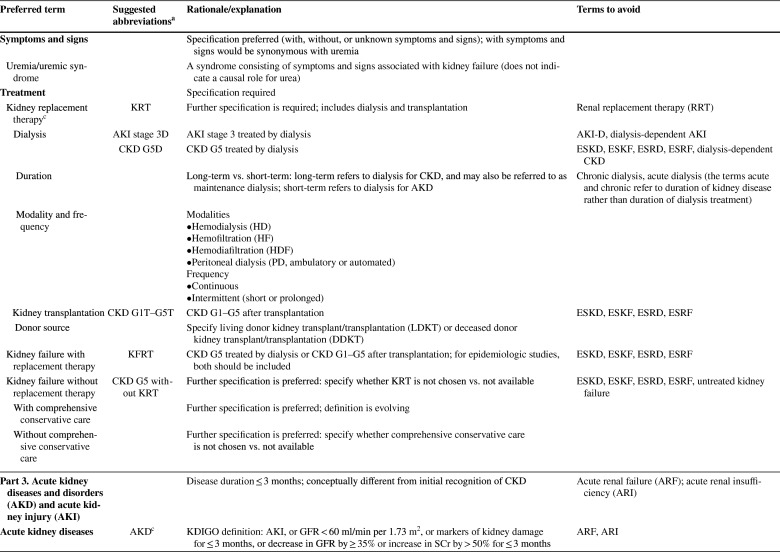

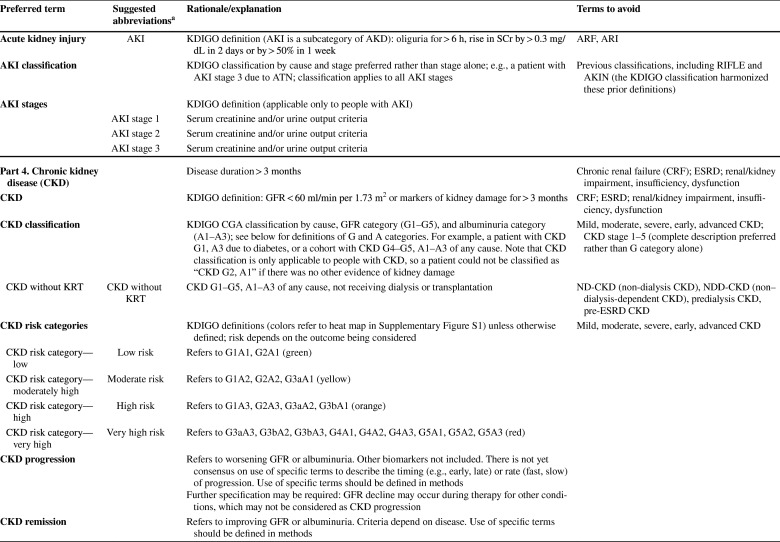

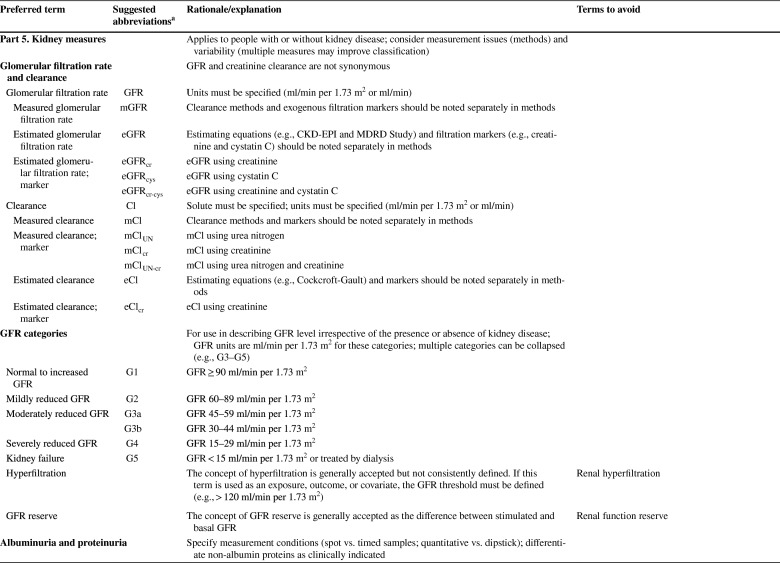

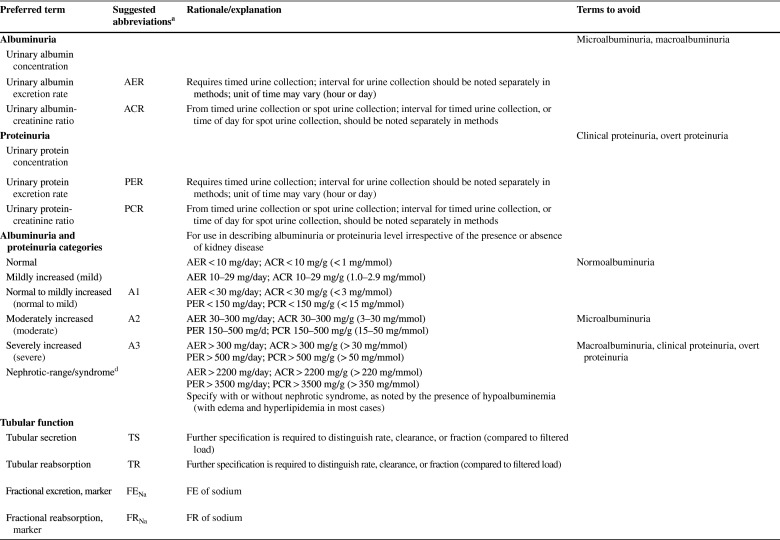
KDIGO Kidney Function and Disease Glossary: suggested terms to describe kidney function and kidney disease, and criteria and measures defining them

ACR, albumin-creatinine ratio; AER, albumin excretion rate; AKD, acute kidney diseases and disorders; AKI, acute kidney injury; AKIN, Acute Kidney Injury Network; ARF, acute renal failure; ARI, acute renal insufficiency; ATN, acute tubular necrosis; CGA, cause, GFR category, and albuminuria category; CKD, chronic kidney disease; CKD-EPI, CKD Epidemiology Collaboration; DDKT, deceased donor kidney transplant/transplantation; eGFR, estimated glomerular filtration rate; ESKD, end-stage kidney disease; ESKF, end-stage kidney failure; ESRD, end-stage renal disease; ESRF, end-stage renal failure; FE_Na_, fractional excretion, sodium; FR_Na_, fractional reabsorption, sodium; GFR, glomerular filtration rate; HD, hemodialysis; HDF, hemodiafiltration; HF, hemofiltration; KDIGO, Kidney Disease: Improving Global Outcomes; KFRT, kidney failure with replacement therapy; KRT, kidney replacement therapy; LDKT, living donor kidney transplant/transplantation; MDRD, Modification of Diet in Renal Disease; mGFR, measured GFR; ND-CKD, non-dialysis CKD; NDD-CKD, non–dialysis-dependent CKD; PCR, protein-creatinine ratio; PD, peritoneal dialysis; PER, protein excretion rate; pre-ESRD, pre–end-stage renal disease; RF, renal failure; RIFLE, Risk, Injury, Failure, Loss of kidney function, and End-stage kidney disease; RRT, renal replacement therapy; SCr, serum creatinine; TR, tubular reabsorption; TS, tubular secretion

^a^Journal style will dictate whether and when to abbreviate terms

^b^Ongoing discussion; may be revised by KDIGO AKI guideline update

^c^Ongoing discussion; may be revised by KDIGO AKD consensus conference

^d^Ongoing discussion; may be revised by KDIGO Glomerulonephritis guideline update

A guiding principle for the development of the glossary was patient-centeredness. The Health and Medicine Division of the US National Academies of Sciences defines patient-centered care as “[p]roviding care that is respectful of, and responsive to, individual patient preferences, needs and values, and ensuring that patient values guide all clinical decisions.”[9] One of the 10 general principles recommended for redesign of the health system is: “Knowledge is shared and information flows freely. Patients should have unfettered access to their own medical information and to clinical knowledge. Clinicians and patients should communicate effectively and share information.” In principle, the terms used to describe kidney function and disease should be understandable to all, with acknowledgment of variation in the level of health literacy. Use of multiple terms with similar meaning can lead to confusion, as can use of terms that forecast the future (such as “pre-dialysis”) rather than describe the present. However, convergence of multiple names into an accepted set of terms does require that users of the glossary are willing to accept that labels that have been preeminent historically, and that may be more familiar or memorable even now, should now be superseded [10].

Of equal importance to patient-centeredness in the development of the glossary was precision, which can generally be defined as exactness or accuracy [10]. How medicine is defined and understood is changing rapidly from a descriptive, disease-based categorization in which multiple pathogenetic pathways may be conflated to a mechanism-based categorization that will promote more precise management of clinical problems. The latter approach, in which a molecular profile is added to the clinical and morphologic profile, has already revolutionized diagnosis and treatment in oncology. In nephrology, the ongoing Kidney Precision Medicine Project, funded by the National Institutes of Health, seeks to ethically obtain and evaluate kidney biopsies from participants with AKI or CKD; create a kidney tissue atlas; define disease subgroups; and identify cells, pathways, and targets for novel therapies [11]. As has occurred in oncology, it is anticipated that refinements that result in more precise disease descriptions will be incorporated into current nomenclature for kidney function and disease, rather than replace it altogether. Thus, although the glossary is designed to be consistent with current knowledge and stable enough to remain relevant for the foreseeable future, it is also intended to be sufficiently flexible to accommodate new vocabulary arising with advances in the field.

A central strength of the proposed glossary is that it is based on existing KDIGO definitions, classifications, and nomenclature for acute and chronic kidney disease. In addition, it was developed using the following: a systematic process, including articulation of a clear and transparent rationale (patient-centeredness and precision); capture of stakeholder viewpoints via patient focus groups [12] and a corresponding survey; a period of public comment on conference scope; and attainment of consensus among attendees at the conference. Although the recommendations are not likely to answer all concerns, the consensus among conference attendees was that standardizing scientific nomenclature is a necessary first step to improving communications among clinicians, researchers, and public health officials, and with patients, their families and caregivers, and the public.

Limitations of the proposed glossary are that it is restricted to English (nuances may be difficult to translate); only a limited number of stakeholders were able to participate, owing to practical reasons; it is not comprehensive (it does not include disease classification, dialysis, transplantation); and further specification is required for studies in children. For these and other reasons, we consider the current recommendations for a glossary to be an important starting point, and it will require future expansion and updating.

Achieving consensus among conference attendees, and publication of the conference report and glossary, is only the first step in implementation of a revised nomenclature. The glossary will be freely available on the KDIGO website (https://kdigo.org/conferences/nomenclature/; Table 2). Elements of the glossary will be included in online updates to the newly released (11th) edition of the *AMA Manual of Style* [13]. Medical journals adopting the recommendations will need to determine how to implement them, and this process will require education of editorial staff as well as proactive communication with authors, generally and with regard to specific manuscripts. If successful, further implementation in clinical practice, research, and public health will require more widespread dissemination and professional education. Improving communication with patients and the public will require efforts to improve patient education and health literacy for the public, and guides to communication with patients. Professional societies, industry, and patient advocacy organizations will be critical to these efforts.

Advances in research, particularly in precision medicine, will introduce a myriad of new terms and novel concepts requiring incorporation into disease definitions and classifications. In addition, the increasing prominence and participation of patient and caregiver communities in defining research and best practices in clinical care will further elucidate the characteristics of patient-centered terminology. Expanding and updating the KDIGO glossary can be accomplished as part of the activities of future KDIGO guideline workgroups and conferences.

## Electronic supplementary material

Below is the link to the electronic supplementary material.

Supplementary file1 (PDF 115 kb) Figure S1. Chronic kidney disease nomenclature used by KDIGO.
