# Supplementary material for: Nomenclature for kidney function and disease: executive summary and glossary from a Kidney Disease: Improving Global Outcomes (KDIGO) consensus conference
Source: J Nephrol. 2020 Jul 16;33(4):639–48. doi: 10.1007/s40620-020-00773-6 (PMC7381458; doi:10.1007/s40620-020-00773-6)
Supplement: Supplementary file 1 — Supplementary file1 (PDF 115 kb) Figure S1. Chronic kidney disease nomenclature used by KDIGO. [file 40620_2020_773_MOESM1_ESM.pdf]

Figure S1. Chronic kidney disease nomenclature used by KDIGO

CKD is defined as abnormalities of kidney structure or function, present for >3 months, with implications for health. CKD is classified based on cause, GFR category (G1–G5), and albuminuria category (A1–A3), abbreviated as CGA.

Prognosis of CKD by GFR and Albuminuria Categories: KDIGO 2012

				Persistent albuminuria categories Description and range		
				A1	A2	A3
				Normal to mildly increased	Moderately increased	Severely increased
				< 30 mg/g<br>< 3 mg/mmol	30-300 mg/g<br>3-30 mg/mmol	> 300 mg/g<br>> 30 mg/mmol
GFR categories (ml/min/ 1.73 m^2) Description and range	G1	Normal or high	≥ 90			
	G2	Mildly decreased	60-89			
	G3a	Mildly to moderately decreased	45-59			
	G3b	Moderately to severely decreased	30-44			
	G4	Severely decreased	15-29			
	G5	Kidney failure	< 15			

Green: low risk (if no other markers of kidney disease, no CKD); Yellow: moderately increased risk; Orange: high risk; Red, very high risk.
